# Effectiveness and safety of low-concentrated ozonized water for the reduction of contamination in dental unit water lines

**DOI:** 10.1016/j.heliyon.2019.e02306

**Published:** 2019-08-21

**Authors:** Keisuke Okubo, Takashi Ito, Yasuyoshi Shiota, Yusuke Kawata, Tadashi Yamamoto, Shogo Takashiba

**Affiliations:** a Department of Pathophysiology - Periodontal Science, Okayama University Graduate School of Medicine, Dentistry and Pharmaceutical Sciences, 2-5-1 Shikata-cho, Kita-ku, Okayama, 700-8525, Japan; b Division of Dentistry, Tottori Municipal Hospital, 1-1 Matoba, Tottori, 680-8501, Japan; c Center for Innovative Clinical Medicine, Okayama University Hospital, 2-5-1 Shikata-cho, Kita-ku, Okayama, 700-8525, Japan; d Division of Dentistry, National Hospital Organization Shikoku Cancer Center, 160 Minamiumemoto-cho-ko, Matuyama, 791-0280, Japan

**Keywords:** Materials science, Dental chair unit water line (DUWL), Biofilm, Heterotrophic bacteria, Ozonized water, Low-concentration

## Abstract

Contamination of dental unit waterlines (DUWL) with heterotrophic bacteria can cause problems in immune compromised patients (aged, tumor and organ transplantation-patients). We focused on the use of low-concentrated ozonized water (OZW) as the biofilm formation restraint system for DUWL. Here, we examined the effects of low-concentrated OZW on the growth of bacteria and related biofilm formation and harmfulness to dental unit components (DUCs) *in vitro*.

**Objectives:**

To evaluate the bactericidal effects of OZW on biofilms in DUWL and DUC *in vitro*.

**Methods:**

Low-concentrated OZW (0.4 mg/L) was generated using an OZS-PTDX generator. Heterotrophic bacterial biofilms in old DUWL tubes and *Candia albicans* solution (control microbe) were treated with OZW for 1 h with gentle agitation before static culturing for 96 h in Reasoner's 2A liquid media. The control solutions were 0.1% cetylpyridinium chloride (CPC), chlorinated tap water (TW), and phosphate-buffered saline (PBS). Adenosine triphosphate (ATP) amounts of the microbes were measured and the biofilms of these microbes were observed using scanning electron microscopy (SEM). Moreover, surfaces of DUC soaked in OZW and TW were observed by SEM.

**Results:**

The OZW reduced ATP levels in microbes to 50% compared to TW and PBS treatment, although CPC reduced it below detection limits. SEM observation revealed deformation of microbes cultured with OZW, whereas no changes were seen on DUC surfaces.

**Conclusions:**

Low-concentrated OZW is bactericidal against heterotrophic bacteria biofilms and it is not harmful to DUC, suggesting that it might be useful in preventing DUWL contamination.

## 1 Introduction

In the recent years, prevention of hospital infections in the elderly and immune compromised hosts has been recognized as a serious problem. Bacterial contamination of dental unit water lines (DUWL) has emerged as a problem, especially in the United States [1]. Although it is essential to use water during dental treatment (for water cooling during preparation of teeth using air turbine hand-piece, for washing and rinsing the mouth), the water coming through DUWL is contaminated with bacteria and causes environmental contamination in dental clinics [2, 3, 4]. In the 1960s, the first report on water contamination through dental units (DU) was published [5, 6]. However, this case was due to contamination of water by oral bacteria that were sucked into the hand-piece because of the negative pressure generated when it was stopped [7]. In fact, some of the microorganisms (*Candida albicans*, etc.) in the oral microbial flora were detected in the water collected from DUWL [4, 8].

In addition to the oral microorganisms getting accidentally sucked and contaminating DUWLs, biofilms were reported as a new source of contamination in the 1990s, as reviewed by O'Donnell [6]. This was caused by the heterotrophic bacteria in water supplied to DUWL. During the closure of clinics (after the end of practice or on doctor's non-consultation days), water supply is stopped for a long period, allowing the bacteria in DUWL to grow and form biofilms [6], even though the supplied water is tap water (TW) chemically disinfected as per regional regulations. The diameter of DUWL tubes is small, which reduces the velocity of water flow near the inner walls compared to that at the center of the tube. This is widely understood as the reason why biofilms are easily formed in DUWL [6, 9]. After the biofilm is thickened, some parts of the biofilm are desquamated and released in hand-piece water. It is reported that the degree of contamination in DU water is high, reaching approximately 10^4^–10^7^ cfu/mL at the first clinical activity of the day [3], although the allowed upper limit of heterotrophic bacteria in drinking water is set at 5 × 10^2^ cfu/mL by Centers for Disease Control and Prevention (CDC), the United States. Opportunistic pathogens such as *Pseudomonas* and *Acinetobacter* were also detected in DU water [10]. Thus, water contamination with oral bacteria sucked-back and heterotrophic bacteria in TW supplied to DUWL potentially causes opportunistic infections in immunocompromised hosts [10].

In addition to the recommendation by the CDC, the acceptable limit of heterotrophic bacteria according to the American Dental Association (ADA) is less than 500 cfu/mL in DUWL water [1]. Furthermore, the organizations recommend the use of sterile water/saline solution for surgical treatments and change of DUWL at appropriate intervals [11]. However, it is difficult to apply these recommendations in regular and daily dental practice. The most popular currently used countermeasures against DUWL water contamination are preventing the back-flow of oral bacteria into hand-piece equipment by using valves and discharging DUWL water before the first treatment of the day (flushing). Although valves for preventing back-flow work efficiently and flushing reduces the number of microorganisms to some extent, it is not possible to entirely remove biofilms [11]. To remove the biofilms adhered on the inner surface of DUWL, a chemical cleaning method, called periodic shock treatment, is performed [12, 13]. However, this method seems to be not widely accepted because the chemicals involved have some risk of affecting the various components of the DUWL. The chemicals used would be also toxic to human cells. Thus, thorough and meticulous washing is the first choice for pollution control before using DU for treatment. Because of the balance between these advantages and disadvantages, chemical maintenance to suppress biofilm formation by bactericidal activity is replacing the periodic shock treatment method. However, it still requires supplying chemicals to the DUWL system. The issues to be handled include safe handling of chemicals and the maintenance of constant supply, especially in developing countries.

Therefore, an easily-handled inexpensive system to suppress biofilm formation is desirable for any general dental clinic. We selected low-concentrated ozonized water to develop a system for the prevention of microbial contamination in DUWL. This system has been used for sanitary and washing purposes of companion animals, since low-concentration ozone disappears after a short time. Ozone exists at about 0.005 ppm in the atmosphere [14], and has a strong oxidizing effect. It has been applied at high concentrations in various fields such as disinfection, bleaching, sterilization, and deodorization [14]. Furthermore, it is utilized in medical field for the prophylaxis and treatment of nosocomial infections [15, 16, 17, 18, 19]. In contrast, ozonized water, that is, the water in which ozone has been dissolved exhibits bactericidal effects even at lower concentrations by producing other reactive oxygen species in water [20]. Equipment for generating low-concentrated ozonized water has been developed for companion animal care. It generates ozone in a simple and safe manner from oxygen in the air and directly bubbles water with ozone, producing low-concentrated ozonized water. Furthermore, this generator has been miniaturized to make it portable for usage in companion animal care. This is an advantage because the equipment can be easily mounted in a regular DU. Therefore, we assumed that equipping the DU with this ozone generator could maintain DUWL with lower levels of contamination by heterotrophic bacteria.

Based on the above background, the aim of this study was to investigate the bactericidal effects of low-concentrated ozonized water on microorganisms and biofilms involved in DUWL contamination and their harmfulness to the components of DUWL.

## 2 Materials and methods

### 2.1 Water and chemicals

Ozonized water was generated from TW using a small generator, OZS-PTDX (Sakuragawa Pump, Osaka, Japan). The ozonized water generators generate ozone by surface discharge control technology [21] and mix ozone into the water using the ejector system. TW (Water Works Bureau at Okayama City, Japan) and phosphate-buffered saline (PBS: Gibco, USA, pH 7.4) were used as negative controls. Aqueous 0.1 % cetylpyridinium chloride solution (CPC: Sigma-Aldrich, St. Louis, MO, USA) was used as the positive control.

### 2.2 Microorganisms

#### 2.2.1 Fungus

*Candida albicans* ATCC 10231 strain (ATCC, USA) was aerobically cultured in brain heart infusion (BHI: Becton, Dickinson and Company, Sparks, MD, USA) liquid media at 37 °C for 12 h. For later experiments, *C. albicans* was re-suspended in test water at 10^8^ cfu/mL.

#### 2.2.2 Heterotrophic bacteria

Planktonic heterotrophic bacteria were obtained from water in a three-way syringe of DU (DUWL-W). For morphological observation, both 50 mL of the mixture (DUWL-W: OZW = 1:9) and 5 mL of DUWL-W were shaken gently and in parallel at 25 °C for 1 h, then 5 mL of each solution was filtered using a nano-percolator/SEM pore (Japan Electronic, Tokyo, Japan).

Biofilms of heterotrophic bacteria were obtained from the inner surface of DUWL tube (DUWL-T) previously used for turbine hand-pieces of DU. DUWL-T was provided for this study in wet conditions after exchanging for a new one in the DU. After DUWL-T was cut by 1-cm length and divided vertically into 2 pieces, 6 pieces of 3-cm length were soaked in 30 mL of the test water and gently shaken at room temperature for 1 h. Bacteria present in the DUWL-T were cultured in Reasoner's 2A (R2A) liquid medium (Wako Pure Chemical Industries, Ltd., Osaka, Japan) at 32 °C for 96 h. Heterotrophic bacteria grown from biofilms were suspended in the media by vigorous mixing.

### 2.3 DUWL components

The components of DUWL used in this study (Table 1) were provided from Morita Manufacturing Sales Department Technology Planning Section 2 (Osaka, Japan). These were new and representative components of DUWL built in the DUs supplied by this company. These components were soaked in either TW or OZW and replaced with fresh ones once a day for six months (only weekdays).

**Table 1:** DUWL components.

Name	Common name	Material
Coupling 1	Brass	C3604BD
Coupling 2	Stainless steel	SUS304
O-ring	Nitrile rubber	NBR
Fluorine-processed tube		PVC resin
Urethane tube		PU (Ether type)

C: JIS standard name of brass (copper alloy).

3604: numbers indicating the family of alloying components i.e. free cutting brass.

BD: bar drawing.

SUS: steel use stainless.

304: numbers indicating steel type symbols conforming to AISI (American Iron and Steel Institute) Standards.

NBR: acrylonitrile-butadiene rubber.

PVC resin: polyvinyl chloride (outside) + polyvinylidene difluoride (inside).

PU: polyurethane.

### 2.4 Measurement of ozone concentration

Ozone concentration was colorimetrically measured using the diethyl-p-phenylenediamine (DPD) method [22] (ozone meter O3-1Z, Kasahara Chemical Instruments Corp., Saitama, Japan). DPD was used as the detection substrate, since it reacts with ozone and develops a color. A 10 mL sample of water in a 15-mL centrifuge tube was mixed with the detection substrate for 1 min and the coloration was compared to the pre-fixed color standards.

### 2.5 Measurement of the amount of adenosine triphosphate (ATP)

The amount of adenosine triphosphate (ATP) present in the bacteria was used to test the vital activity of bacteria. ATP amount was measured using a Lucifer HS kit and Lumi-tester C-110 (Kikkoman Bio-Chemiphar, Tokyo, Japan), which employs luciferin-luciferase reaction [23]. The amount of luminescence was recorded as the values of relative light unit (RLU, detection range: 1.0 × 10^−16^ to 3.0 × 10^−11^ mol of ATP). Background ATP present outside the bacterial cells was removed using adenosine phosphate deaminase.

### 2.6 Observation by scanning electron microscopy (SEM)

The samples observed by SEM were *C. albicans* treated with each test water sample, planktonic heterotrophic bacteria in DUWL filtered by nano-percolator/SEM pore, biofilms on the inner surface of used DUWL-T, and unused DUWL components made of brass, stainless steel, ethylene-acryl-nitrile rubber, polyurethane, and polyvinylidene difluoride.

DUWL-T was immersed in 3-methyl-butyl for 1 h as a replacement after dehydration with an ascending ethanol series (50, 70, 90, 95%, and absolute ethanol), and it was sublimated with the critical point dryer (JCPD-5: Jeol, Tokyo, Japan). These steps were omitted for other samples.

The surfaces of each sample were treated with osmium by vapor deposition method using a vacuum evaporator (Neoc-osmium coater: Meiwafosis, Tokyo, Japan). The morphology of the microorganisms and surface condition of each component was observed using a field emission scanning electron microscope (FE-SEM DS-720: Topcon, Kyoto, Japan; accelerating voltage 15 kV).

### 2.7 Statistical analysis

Statistical processing was performed using Student's *t*-test (Microsoft Excel) after using an F-test (Microsoft Excel) to analyze the variance between groups. *P*-values less than 0.05 were considered statistically significant.

## 3 Results

### 3.1 Ozone concentration in OZW

**Fig. 1:**
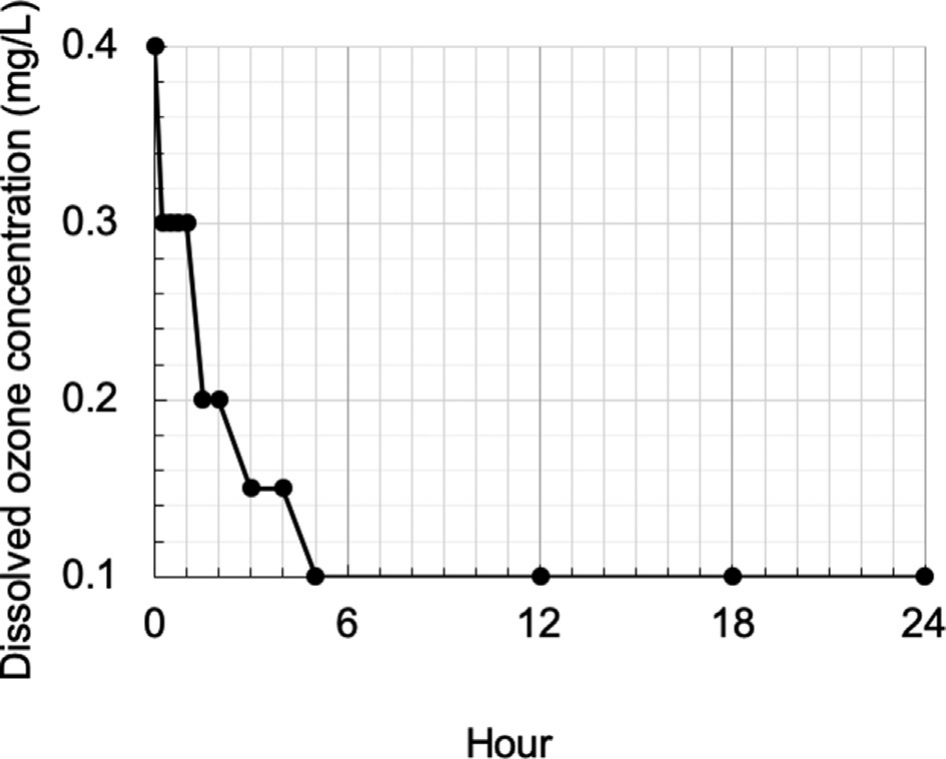
Time-dependent changes in dissolved ozone concentration in OZW. The changes in ozone concentration of OZW (10 mL) sampled from an ozone generator (OZS-PTDX) were measured using DPD colorimetry. Data are the average values of three independent experiments (N = 3). Each experiment was performed in triplicate. Standard deviations are not indicated because all the measurements had the same values.

The ozone concentration in OZW changed over time (Fig. 1), with a maximum concentration of 0.4 mg/L immediately after sampling. Subsequently, it reduced to half within 2 h, and further decreased to the lowest concentration of 0.1 mg/L by 5 h. This final concentration was equivalent to that of TW.

### 3.2 Planktonic *C. albicans* was sensitive to OZW

**Fig. 2:**
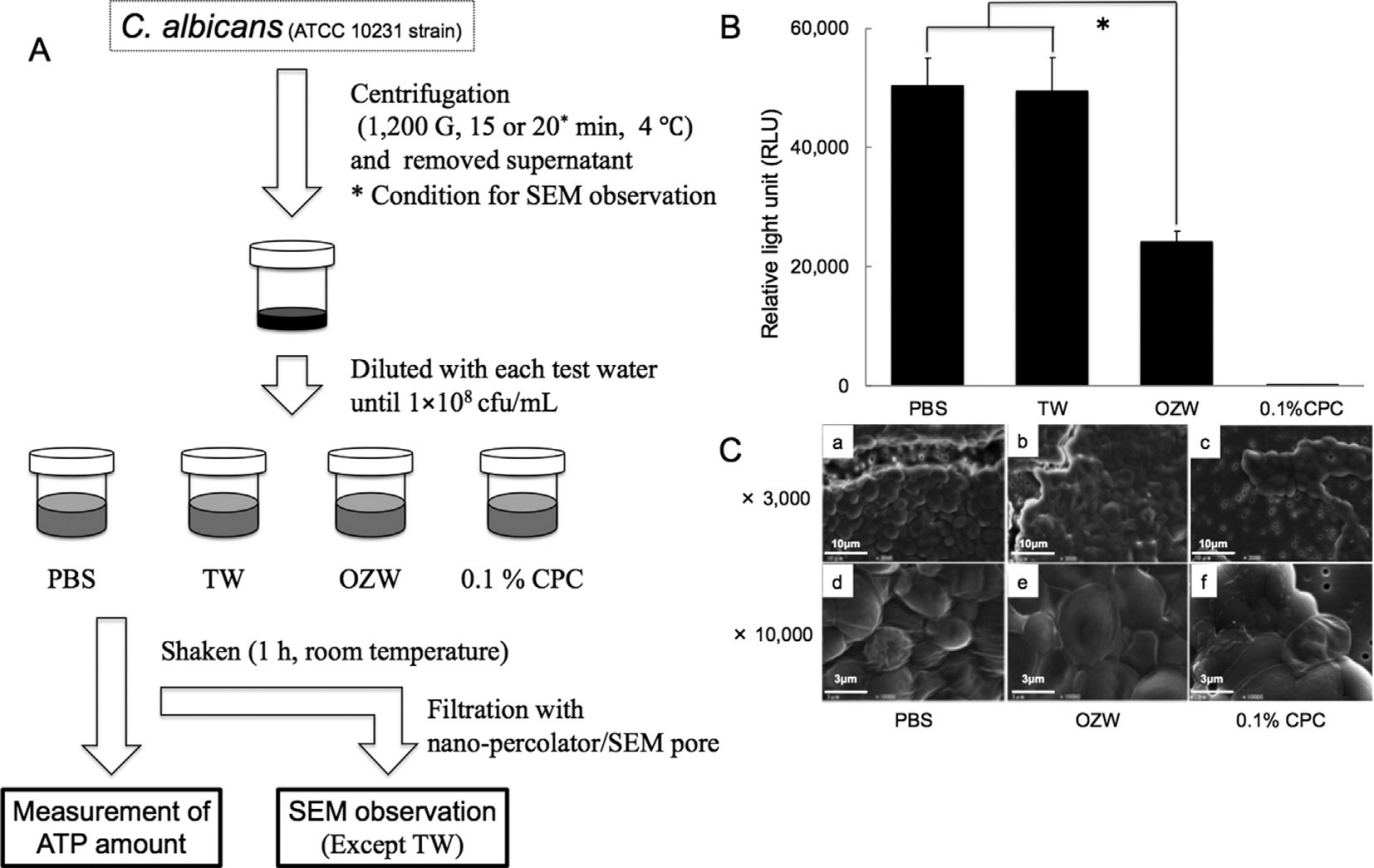
Bactericidal activity against cultured planktonic *C. albicans* (A) The schematic sketch of this examination This schematic sketch indicate the concrete method and procedure in this experiment (B) Change in ATP amount *C. albicans* was treated with test water (10^8^ cfu/mL) for 1 h. After centrifugation, ATP amount of *C. albicans* was measured by the luciferin-luciferase reaction method. Data are the average values of three independent experiments. Each experiment was performed in triplicate (N = 3). Error bars indicate standard deviation. ∗, *p* < 0.05 (C) Morphological changes *C. albicans* treated as mentioned above was filtered and observed under SEM. Panels are representative images of 2 independent specimens (N = 2). Scale bars: 10 μm in x 3,000; 3 μm in x 10,000.

ATP amounts of *C. albicans* treated with OZW reduced to half of the negative controls (PBS and TW; Fig. 2B) after treatment for 1 h, and no ATP was detected after treatment with 0.1 % CPC solution. *C. albicans* treated with PBS had clear margins and spherical forms. Filiform spore-like structures were observed among the cells (Figure 2C-a, -d). However, *C. albicans* treated with 0.1% CPC had unclear margins and the forms were elliptical and flattened (Figure. 2C-c, -f). On the other hand, *C. albicans* treated with OZW retained the characteristics of both conditions, slightly resembling positive control with swelling of bodies and ambiguity of margins (Fig. 2C-b, -e).

### 3.3 Planktonic microorganisms in DUWL-W were sensitive to OZW

**Fig. 3:**
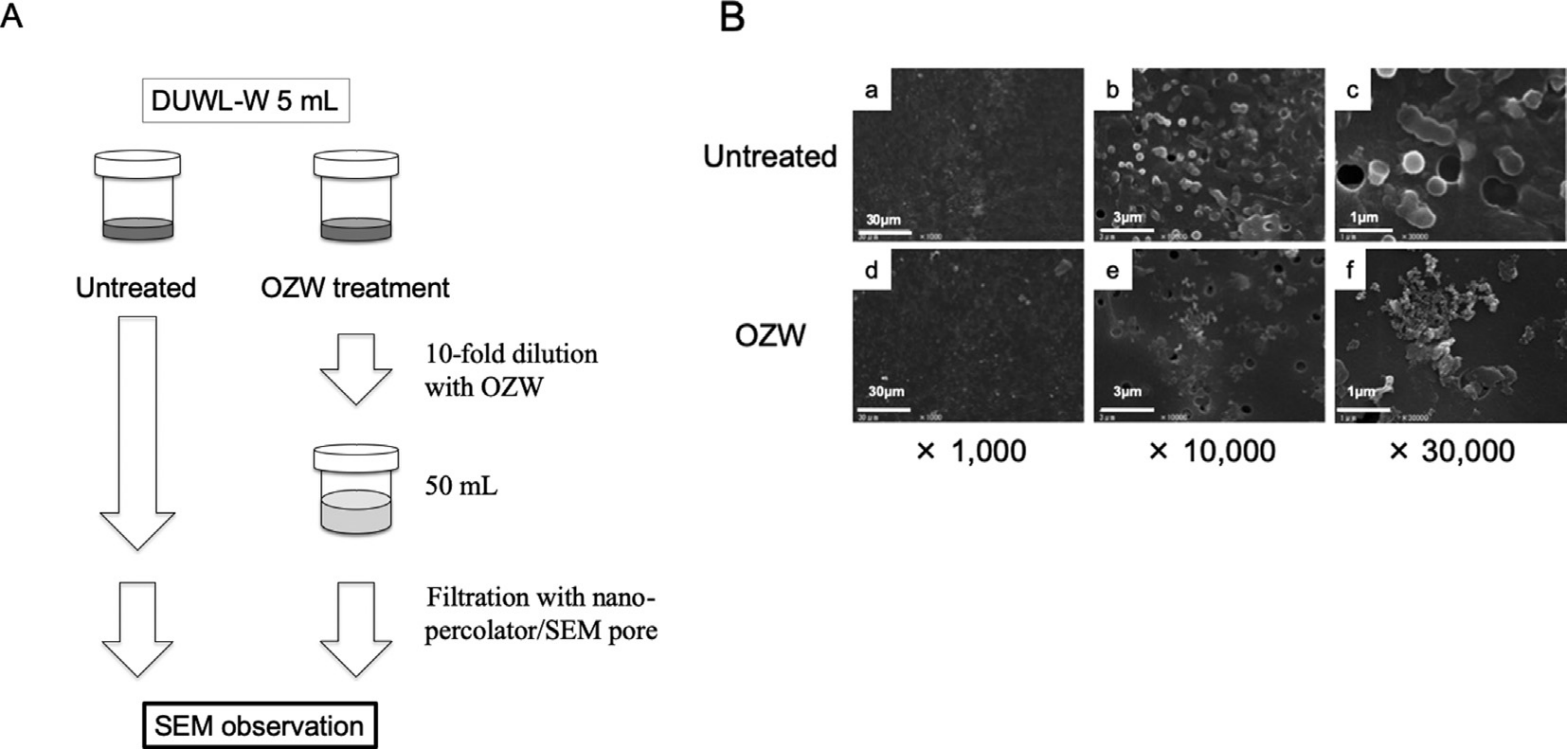
Bactericidal activity against planktonic heterotrophic bacteria in DUWL-W (A) The schematic sketch of this examination The schematic sketch indicates the concrete method and procedure in this experiment (B) Morphological changes Planktonic heterotrophic bacteria obtained from DUWL-W in a three-way syringe were treated using OZW for 1 h. Bacteria were filtered and observed under SEM. Panels are representative images of 2 independent specimens (N = 2). Scale bars: 30 μm in x 1,000; 3 μm in x 10,000; 1 μm in x 30,000.

Spherical microorganisms were observed in DUWL-W (Fig. 3B-b, -c). However, no spherical microorganisms were observed after DUWL-W was mixed with OZW and treated for 1 h (Fig. 3B-e, -f). Only debris-like residues were observed.

### 3.4 DUWL biofilms were sensitive to OZW

**Fig. 4:**
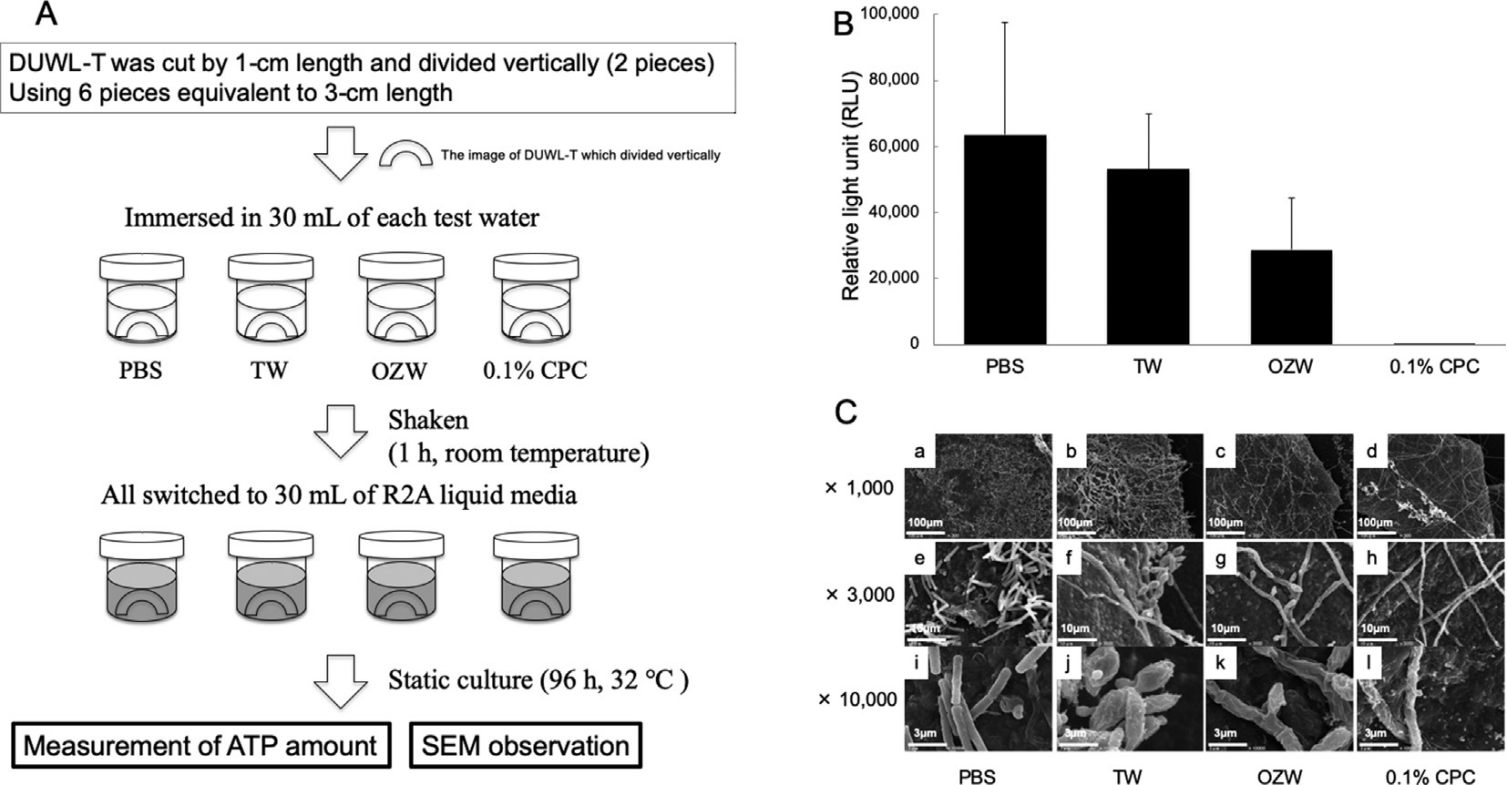
Bactericidal activity against biofilms in DUWL-T (A) The schematic sketch of this examination. The schematic sketch indicates the concrete method and procedure in this experiment (B) Changes in ATP amount DUWL-T was treated with test water for 1 h. After the test water was removed, DUWL-T was incubated in R2A liquid medium for 96 h. ATP amount of suspended heterotrophic bacteria was measured by the luciferin-luciferase reaction method. Data are the average values of 4 of 6 measurements from a single experiment (N = 2). Maximum and minimum data were excluded for the average. Error bars indicate standard deviation (C) Morphological observation Heterotrophic bacteria were grown as mentioned above, but vigorous mixing was not performed. The samples were observed using SEM. Panels are representative images of 2 independent specimens (N = 2). Scale bars: 100 μm in x 300; 10 μm in x 3,000; 3 μm in x 10,000.

ATP amounts of DUWL biofilms were reduced by OZW, but it was not statistically significant after treatment for 1 h (Fig. 4B). No ATP amounts were detected in those treated with 0.1 % CPC solution. When DUWL biofilms were treated with each solution, the density and morphology of microorganisms comprising the biofilm varied. Microorganisms treated with PBS were homogeneous and had clear margins (Fig. 4C-a, -e, -i). However, microorganisms treated with TW, OZW, and 0.1% CPC solution seemed to be deformed (Fig. 4C-b, -f, -j), deflated (Fig. 4C-b, -f, -j), and withered (Fig. 4C-c, -g, -k), as tending to be severely deformed. In addition, the density of bacteria on the biofilm surface decreased in the same order.

### 3.5 DUWL components were not sensitive to OZW

**Fig. 5:**
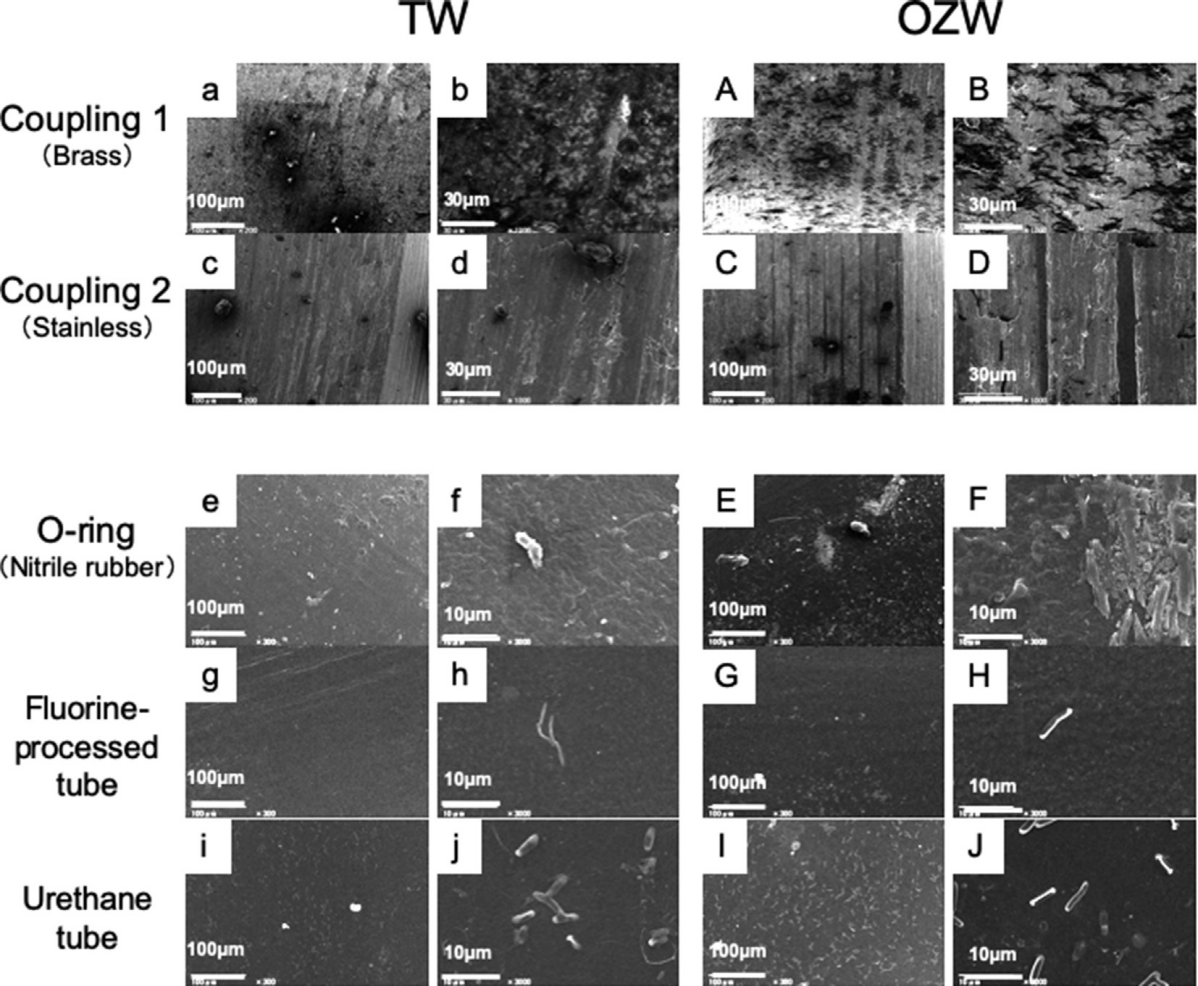
Influence on DUWL components. Components of DUWL were repeatedly immersed in TW or OZW once a day for 6 months, then used for SEM observation. Panels are representative images of 2 independent specimens (N = 2). Scale bars: 100 μm in x 200 or 300; 30 μm in x 10,000; 10 μm in x 3,000.

No significant surface irregularities, including roughness, projection, recess, corrosion, or distortion, were observed by SEM after the DUWL components were soaked in either OZW or TW for 6 months (Fig. 5). However, some bacilli (approximately 10 μm length) were observed on the surfaces of O-rings, fluorine-processed tubes, and urethane tubes.

## 4 Discussion

In the present study, we examined the effectiveness and safety of low-concentrated ozonized water in preventing bacterial DUWL contamination. According to the results, ozonized water at a maximum concentration of 0.4 mg/L had bactericidal effects on the microorganisms involved in DUWL contamination. Biofilms already formed in DUWL-T remained on the surface of the tube, but the morphological characteristics of biofilms and bacteria changed, suggesting damage to the biofilms. Furthermore, no morphological changes in the DUWL components were observed.

At the beginning of this study, we determined the time-dependent change of dissolved ozone concentration in OZW generated from an ozone generator (Fig. 1). The concentration of ozone decreased from the highest level (0.4 mg/L) to TW background level (0.1 mg/L) within 5 h. The maximum concentration of ozonized water was 0.4 mg/L, which was lower than that of the sterilizing application used in a previous study [24]. Moreover, the dissolved ozone concentration was immediately reduced after sampling. Based on the decline curve (Fig. 1), the period for which the concentration is effective may be 1 h, although the half-life of ozonized water is 2 h. Consequently, subsequent experiments were designed with a reaction time of 1-hour.

To test the bactericidal effects of OZW on planktonic microorganisms, we used *C. albicans* (Fig. 2A) and heterotrophic bacteria in the water from a three-way syringe (DUWL-W). There were two reasons for choosing *C. albicans* to represent the planktonic microorganisms. First, *C. albicans* is a popular pathogenic contaminant, and it has already been reported as one of the microorganisms present in DUWL water [4, 8]. Second, fungi have stronger cell walls than bacteria [25], making them more resistant to OZW than bacteria. We found that OZW reduced the ATP amounts by half (Fig. 2B), and the morphology of *C. albicans* treated with OZW resembled the deformed morphology after treatment with CPC (Fig. 2C). The latter finding was consistent with the previous report on the disinfection mechanism of ozonized water [26]. As reported by Arita, viable *C. albicans* cells were nearly nonexistent after exposure to flowing ozonized water (2 or 4 mg/L) for 1 min [24]. In our results, the vital activity was reduced by only half, as the maximum ozone concentration from our ozone generator was lower than their equipment.

Heterotrophic bacteria from three-way syringe were also in planktonic conditions, mainly detached from biofilm formed in DUWL. When DUWL was used for heterotrophic bacterial culture on R2A agar plate before this study, more than 10^8^ cfu/mL of bacteria were observed (data not shown). Heterotrophic bacteria in 5 mL of DUWL were filtered and visualized under SEM (Fig. 3B-a, -b, -c). After mixing the DUWL with OZW at a 5:1 ratio, these bacteria deformed, shrank, and their residues were observed. This finding suggests that heterotrophic bacteria were damaged by OZW treatment. Thus, the bacteria were disrupted after filtration.

We confirmed the bactericidal effect on biofilms formed by heterotrophic bacteria inside DUWL. Because biofilms consist of layered structures [27], some bacterial species adhering to the tube surface (bottom of biofilm) cannot enter the DUWL-W, but other bacterial species inhabiting the surface layer of the biofilm can easily enter DUWL-W. Therefore, we investigated the OZW effect on both DUWL-W and DUWL-T. We confirmed the bactericidal effect on microorganisms contained in DUWL-W (Fig. 3B-e, -f). In DUWL-T, we found that OZW reduced ATP amounts of the microorganisms in biofilms by half compared to negative controls, but the effect was not significant (Fig. 4B). OZW seemed to cause morphological changes, its effect was less than that of CPC (Fig. 4C-c, -g, -k).

These results collectively suggest that OZW may reduce the growth of heterotrophic bacteria in DUWL through continuous treatments. However, the application of OZW may be limited to unused DUWL-T, because it is unclear if OZW can destroy biofilm structure.

Finally, we determined the effect of OZW oxidation on main DUWL components. The DUWL components seemed to tolerate low-concentrated OZW. Significant deterioration and abnormal findings on the surface were not observed either by naked eye inspection or SEM (Fig. 5). However, there were some microorganisms on the sample's surface. This may be from contamination during the long-term tests, but fewer microorganisms were found on the surface of the fluorine-processed tubes than on the urethane tubes. Thus, it is also important to consider the condition of the tube surfaces.

The ozonized water used in this study could reduce the growth of microorganisms without apparently harming DUWL tubes. The advantages are that there is little risk of the tube deteriorating and organic substances eluting due to the strong oxidizing power of ozone, and that microorganism adhesion is reduced due to the smoothness of the tube surface. Thus, it is reasonable to consider that the risk of unknown adverse effects for patients from using low-concentrated ozonized water is low.

## 5 Conclusions

Low-concentrated OZW at 0.4 mg/L is bactericidal to microorganisms involved in DUWL contamination without obvious damage to the components of DUWL. Therefore, low-concentrated OZW can be used to prevent bacterial contamination in DUWL. However, the limitations of this study include the use of static conditions for DUWL using low concentrations of OZW.

Further studies using simulated DUWL with periodical clinical conditions over a long period are needed.

## Clinical significance

Low-concentrated OZW demonstrated significant advantages—it effectively reduces bacterial biofilm contamination and causes less damage to DUC. Therefore, establishment of the ozonized water supply system could be a promising solution for decontamination in DUWL and may reduce the risk of hospital-acquired infections in dental practice.

## Declarations

### Author contribution statement

Keisuke Okubo: Conceived and designed the experiments; Performed the experiments; Analyzed and interpreted the data; Contributed reagents, materials, analysis tools or data; Wrote the paper.

Takashi Ito: Conceived and designed the experiments; Analyzed and interpreted the data.

Shogo Takashiba: Conceived and designed the experiments; Analyzed and interpreted the data; Wrote the paper.

Yasuyoshi Shiota: Performed the experiments; Analyzed and interpreted the data; Contributed reagents, materials, analysis tools or data.

Yusuke Kawata: Performed the experiments; Contributed reagents, materials, analysis tools or data.

Tadashi Yamamoto: Contributed reagents, materials, analysis tools or data; Wrote the paper.

### Funding statement

This work was supported by Organization for Research Promotion & Collaboration in Okayama University.

### Competing interest statement

The authors declare no conflict of interest.

### Additional information

No additional information is available for this paper.
